# DDT Exposure of Zebrafish Embryos Enhances Seizure Susceptibility: Relationship to Fetal *p,p*′-DDE Burden and Domoic Acid Exposure of California Sea Lions

**DOI:** 10.1289/ehp.11685

**Published:** 2008-08-01

**Authors:** Jessica A. Tiedeken, John S. Ramsdell

**Affiliations:** Marine Biotoxins Program, Center for Coastal Environmental Health and Biomolecular Research, National Oceanic and Atmospheric Administration—National Ocean Service, Charleston, South Carolina, USA

**Keywords:** *Danio rerio*, DDT, domoic acid, seizures, zebrafish

## Abstract

**Background:**

California sea lions have a large body burden of organochlorine pesticides, and over the last decade they have also been subject to domoic acid poisoning. Domoic acid poisoning, previously recognized in adult animals, is now viewed as a major cause of prenatal mortality. The appearance of a chronic juvenile domoic acid disease in the sea lions, characterized by behavioral abnormalities and epilepsy, is consistent with early life poisoning and may be potentiated by organochlorine burden.

**Objective:**

We investigated the interactive effect of DDT (dichlorodiphenyltrichloroethane) on neurodevelopment using a zebrafish (*Danio rerio*) model for seizure behavior to examine the susceptibility to domoic acid–induced seizures after completion of neurodevelopment.

**Methods:**

Embryos were exposed (6–30 hr postfertilization) to either *o,p*′-DDT or *p,p*′-DDE (dichlorodiphenyldichloroethylene) during neurodevelopment via a 0.1% dimethyl sulfoxide solution. These larval (7 days postfertilization) fish were then exposed to either the seizure-inducing drug pentylenetetrazol (PTZ) or domoic acid; resulting seizure behavior was monitored and analyzed for changes using cameras and behavioral tracking software.

**Results:**

Embryonic exposure to DDTs enhanced PTZ seizures and caused distinct and increased seizure behaviors to domoic acid, most notably a type of head-shaking behavior.

**Conclusion:**

These studies demonstrate that embryonic exposure to DDTs leads to asymptomatic animals at completion of neurodevelopment with greater sensitivity to domoic acid–induced seizures. The body burden levels of *p,p*′-DDE are close to the range recently found in fetal California sea lions and suggest a potential interactive effect of *p,p*′-DDE embryonic poisoning and domoic acid toxicity.

The organochlorine pesticide DDT (dichlorodiphenyltrichloroethane), banned in the United States in the early 1970s after > 20 years of industrial and agricultural discharge, is a persistent pollutant, most notably in the shallow water areas of Palos Verdes shelf in southern California (MacGregor 1976). The persistence of DDT and its more stable metabolite DDE (dichlorodiphenyldichloroethylene) in sediments and the food web and its intense bio accumulation in apex predators is the cause for substantial monitoring and continued concern over potential health effects. The California sea lion, an apex predator with a foraging range of its primary female popu lation within the DDT industrial outfall (Connolly and Glaser 2002), accumulates some of the highest levels of DDT of any species, with values exceeding 1 mg/g blubber wet weight (Le Boeuf and Bonnell 1971). Very high organochlorine levels, including not only DDTs, but polychlorinated biphenyls (PCBs) as well, have been associated with reproductive failure at the primary California sea lion rookery of San Miguel Island (DeLong et al. 1973). Females in this population found with aborted pups in 1970 had 8- and 4-fold higher concentrations of DDTs and PCBs, respectively, than females birthing term pups (Gilmartin et al. 1976). However, the cause–effect relationship of this correlation remained questionable because of the presence of leptospirosis and San Miguel sea lion virus in this population. Over the subsequent 20 years, the DDT burden in the California sea lion has decreased by at least an order of magnitude (Le Boeuf et al. 2002); however, mass reproductive failure is still observed and more recently has been correlated with exposure to the algal toxin domoic acid (Brodie et al. 2006). Analysis of blubber from premature and term sea lion pups and parturient sea lion cows demonstrated that high maternal organochlorine concentrations are transferred to the fetus (Greig et al. 2007). Detailed analysis of the aborted and premature births suggests multiple causes, with domoic acid currently viewed as a major contributing factor (Goldstein et al., in press).

Domoic acid is a tricarboxylic acid produced by certain species of the cosmopolitan diatom *Pseudonitzschia* (Wright et al. 1989). It causes excitotoxicity and damage to neuronal pathways responsible for the learning and recall of sequences underlying spatial memory and restraining seizure-prone circuitry associated with temporal lobe epilepsy (Ramsdell 2007). It is responsible for human poisonings via shellfish consumption (Perl et al. 1990) and the death of marine mammals, especially California sea lions via consumption of planktivorous fish (Scholin et al. 2000). In experimental animals, domoic acid causes abortion in midgestation mice when administered to the dams at near lethal doses (Khera et al. 1994). Domoic acid readily crosses the placenta to accumulate in the amniotic fluid and reaches biologically effective levels in the prenatal brain (Maucher and Ramsdell 2007). It also causes delayed persisting effects in pups when administered at lower doses during pregnancy, enhancing susceptibility to agents that induce seizures and memory loss (Dakshinamurti et al. 1993; Levin et al. 2005). Domoic acid, which is normally cleared rapidly by renal filtration in adult animals, is more toxic to animals *in utero* because of a longer residence time in the fetal-maternal unit and greater access to the fetal brain (Ramsdell and Zabka 2008). We have proposed that embryonic exposure to domoic acid may lead to a different set of consequences than those found in the adult, namely, a disorder resulting in excitable brain manifest in juvenile and adult life (Tiedeken and Ramsdell 2007). Moreover, we proposed that a similar disorder resulting from *in utero* domoic acid toxicity in the California sea lion (Ramsdell and Zabka 2008) may be a cause in the newly recognized chronic disease characterized by altered behaviors and epilepsy (Goldstein et al. 2008).

In the present study we investigated the interactive effects of domoic acid and the DDT congeners *o,p*′-DDT and *p,p*′-DDE on neuro-development using zebrafish. Fish embryos show sensitivity to the effects of *o,p*′-DDT (Edmunds et al. 2000; Ton et al. 2006) and domoic acid (Tiedeken et al. 2005). We used a zebrafish model for pentylenetetrazol (PTZ)-induced seizure behavior (Baraban et al. 2005), modified to examine how embryonic exposure affects susceptibility to chemical-induced seizures after completion of neurodevelopment (Tiedeken and Ramsdell 2007). In this study, we exposed zebrafish embryos to *o,p*′-DDT and *p,p*′-DDE and then investigated larval fish sensitivity first to the well-characterized chemical convulsant PTZ and then to environmental toxin domoic acid. We then correlated the body burden of DDT and DDE of the zebrafish to levels measured in the blubber of fetal sea lions in the early 1970s and 2000s to evaluate the feasibility of an interaction with domoic acid that may cause a fetal origin for juvenile and adult disease.

## Materials and Methods

### Zebrafish

We obtained 15 breeding pairs of AB wild-type strain zebrafish (*Danio rerio*) from Zebrafish International Resource Center (ZIRC) (Eugene, OR) and allowed them to breed at random for embryo production. Fish were kept on a 14-hr light:10-hr dark cycle in a recirculating aquarium rack system (Aquatic Habitats, Apopka, FL). Water conditioning and environmental quality was maintained according to aquarium system use and care manual and *The Zebrafish Book* (Westerfield 2000). Utmost care was used to ensure that the animals were treated humanely, and in cases where distress could not be alleviated, the animals were euthanized. Zebrafish were fed twice daily with Zeigler Adult Zebrafish Diet (Aquatic Habitats), and on afternoons before breeding, the diet was supplemented with live *Artemia*. Breeding inserts were placed in tanks with a plastic plant to collect fertilized eggs (embryos) in the morning. The embryos were removed from the bottom of the tank within the first hour of light and washed with sterile tank water.

### Solution preparation

The organochlorine pesticides *o,p*′-DDT and *p,p*′*-*DDE were resuspended and diluted in DMSO (dimethyl sulfoxide). Working stocks were created at 1,000 times of tested concentrations. These stocks were in half-log concentrations of 1, 3, 10, 30, and 100 mM. In addition, the DDE was diluted to a lower range of concentrations (0.03, 0.1, 0.3, 1 mM) for assistance in range finding.

Both PTZ and domoic acid were used as seizure-inducing agents for the 7 days post-fertilization (dpf) exposure. A concentration of 5 mM PTZ in Ringers solution (Westerfield 2000) was used, as it showed the greatest effect in previous studies (Tiedeken and Ramsdell 2007). Following a range finder, a concentration of 0.36 mM of domoic acid in Ringer’s solution was determined to have a noticeable, rapid, and sublethal effect on the fish.

All chemicals, unless otherwise noted, were purchased from Sigma Chemical Company (St. Louis, MO).

### Embryonic organochlorine exposure

Embryos were staged and allowed to mature to the germ ring/shield stage, occurring around 6 hr postfertilization (hpf). Groups of 80 embryos were placed in 6-well plates (Falcon 35-3046, Becton Dickinson Labware, Franklin Lakes, NJ) with 1 mL sterile zebrafish water (Westerfield 2000) per 10 embryos. For each milliliter in a well, 1 μL of the organochlorine working stock was added and thoroughly mixed, creating a 0.1% DMSO and micromolar organochlorine concentration (Ton et al. 2006). One well contained embryos in media only, and another contained embryos exposed to the vehicle DMSO only for controls. Embryos remained in the organochlorine solution for 24 hr in a 28.5°C incubator. After 24 hr, the embryos were transferred to a new plate with fresh media and raised in the incubator until reaching 7 dpf.

### Larval challenges

At 7 dpf, the organochlorine-exposed embryos were challenged to the seizure-inducing agent PTZ in the exact same manner as reported previously (Tiedeken and Ramsdell 2007). We used a concentration of 5 mM PTZ across all embryo treatments because this level showed the most significant response in the prior study. For analytical purposes, all subjects were euthanized after observations with a gradual exposure to cold temperature.

Challenges to domoic acid were conducted with a similar 96-well plate setup, with domoic acid concentrations replacing those of the PTZ. Subjects were monitored for 20 min after the addition of the toxin.

### Organochlorine body burden

Larval fish 7 dpf were grouped by preexposure in microcentrifuge tubes and stored at –20°C until analyzed. Care was taken to remove all excess water, and wet weight was recorded. For extraction, each sample was transferred to a cell with dried diatomaceous earth and an exact amount of NIST organochlorine deuterated standard (National Institute of Standards and Technology, Gaithersburg, MD). The cell was extracted into dichloro-methane using Accelerated Solvent Extractor (ASE 200; DIONEX, Sunnyvale, CA) equipment. Solvent volume was reduced and replaced with hexane. Samples were run through a 2-g alumina column to remove impurities. Gas chromatography/mass spectrometry was used to determine the quantity of organochlorine in the group and was used to calculate body burden.

### Analysis

We compared organochlorine-induced morphologic and behavioral differences using recorded images from an RGB AutomatiCam camera (A209; Microimage Video Systems, Boyerton, PA) attached to a Leica MZ 12 stereomicroscope (Leica Microsystems Inc., Bannockburn, IL). We reviewed recordings of the larvae taken with the TrackSys Tower (Tracksys Ltd., Nottingham, UK) following the addition of PTZ for behavioral changes and seizures. We also noted the time to reach the first definitive stage II seizure (seizure latency). Distance moved and mobility parameters were obtained from EthoVision tracking program analysis (Noldus Information Technology Inc., Leesburg, VA), and baseline parameters were subtracted prior to statistics. Due to the time-dependent nature of the trials, those individuals that did not track properly were excluded. One-way analysis of variance (ANOVA) followed by a Dunnett’s comparison test (Prism version 4; GraphPad Software Inc., San Diego, CA) were used to analyze the significance of the compounding effects of both the organochlorines and PTZ responses on the larval fish.

Due to the nature of the domoic acid response, the recordings were performed using the RGB camera attached to the scope. Behaviors were scored manually, noting time of occurrence and number of occurrences for each individual. Occurrences were noted when the larvae switched to a different behavior or the behavior endured longer than a minute. Behaviors were grouped by perceived severity and ability to recover from the behavior. Touch response was checked periodically by gently tapping the tail with a polished end of a micropipette.

## Results

### Effect of embryonic exposure to o,p′-DDT and p,p′-DDE on PTZ-induced seizures in larval zebrafish

Zebrafish embryos immersed in DDT between 6 and 30 hpf showed limited adverse effects to the organochlorine after completion of neurodevelopment (7 dpf). Groups of embryos had similar viability, around 95% survival, across all exposures to *o,p*′-DDT except for the largest dose, 100 μM, where 17% of embryos were dead by the end of the acute exposure period (30 hpf). After transfer to a clean environment, low mortality rates (< 5%) were consistent across all groups as they completed neuronal development. Spinal deformity was noted in 86% of the 30-μM group and 95% of the 100 μM DDT-treated newly hatched larvae. Embryos exposed to *p,p*′-DDE experienced < 10% mortality similar to the control groups at 30 hpf. No distinguished deformities were noted between different *p,p*′-DDE exposure levels.

**Figure 1:**
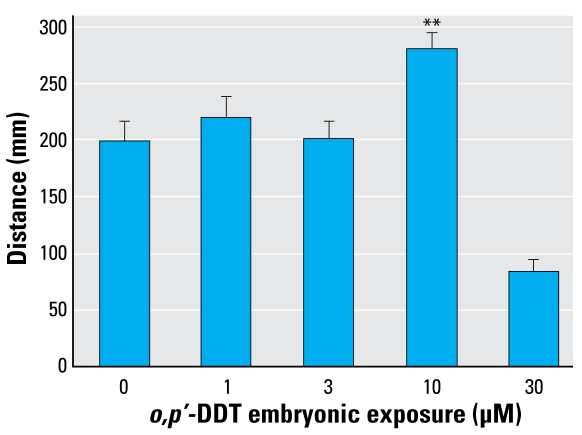
Increase above baseline in total distance traveled (mean ± SE) after a 15-min bath exposure to PTZ across levels of *o,p*′-DDT embryonic exposure. **Distances traveled were significantly greater (*p* < 0.01) than that of vehicle-only treatment.

**Figure 2:**
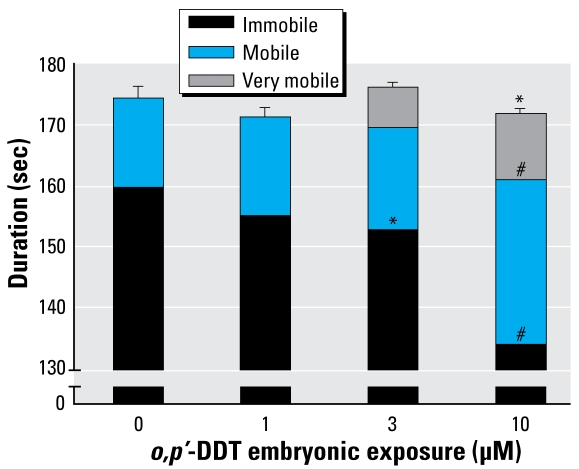
Duration of mobility states of embryonic *o,p*′-DDT–exposed larvae during EthoVision trial (mean ± SE) after exposure to PTZ. *p < 0.05; and ^#^*p* < 0.001 compared with vehicle.

The cohort of zebrafish exposed to 10 μM *o,p*′-DDT as embryos experienced a notable increase in PTZ-induced seizure activity, as shown through increased distance traveled and increased time in a mobile state. (Figures 1 and 2). A smaller effect was seen in the latter indicator at the next lower dose of 3 μM *o,p*′-DDT. Upon exposure to the PTZ, animals in the high *o,p*′-DDT embryonic dose groups (30–100 μM) showing morbidity displayed little overall movement, and few experienced seizure behavior. The time to first marked stage II–type seizure in response to PTZ was not significantly affected with embryonic DDT treatment.

**Figure 3:**
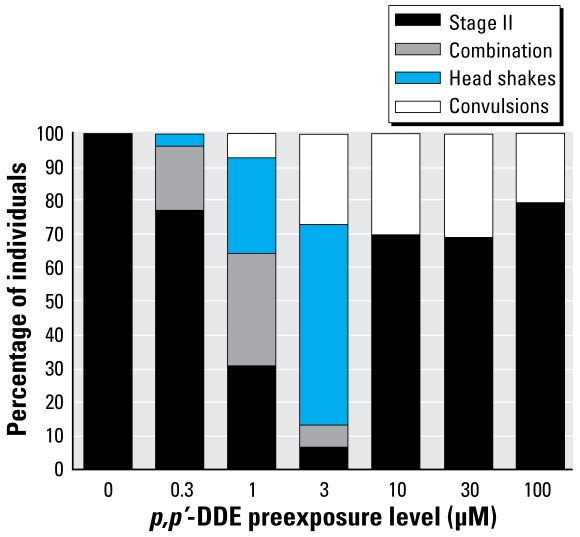
Percent of mobile individuals responding with selected behaviors to 5-mM PTZ exposure at 7 dpf after embryonic exposure to concentrations of *p,p*′-DDE. Combination denotes those that exhibited both a definable stage II seizure along with head-shake behaviors.

**Figure 4:**
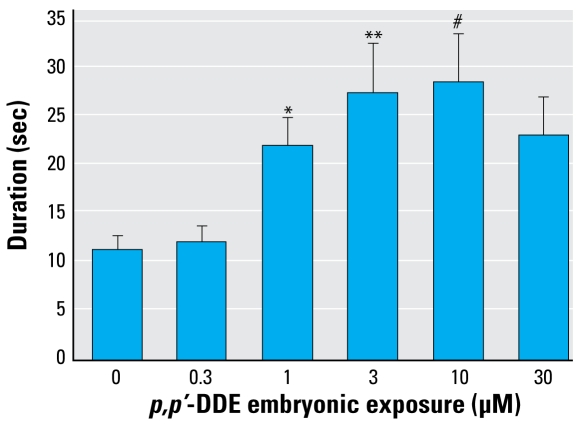
Duration (mean ± SE) of mobile state measured in embryo-exposed *p,p*′-DDE larvae after exposure to PTZ. *p < 0.05; **p < 0.01; and ^#^*p* < 0.001 compared with vehicle.

Zebrafish exposed to 0.3–100 μM *p,p*′-DDE as embryos experienced a head-shake motion in response to PTZ in addition to or in place of seizure behavior (Figure 3). This behavior was unique to this pretreatment and was not identified in vehicle-only (DMSO) or nontreated groups. The head-shake motion coupled with defined seizure behaviors contributed to the increased duration of a mobile state noted in 1–10 μM DDE treatment groups (Figure 4). Because of the variability in individual responses across the DDE treatment groups, differences in distance traveled was not a useful measure, as it was for DDT.

### Effect of embryonic exposure on DDT body burden of larval zebrafish

**Figure 5:**
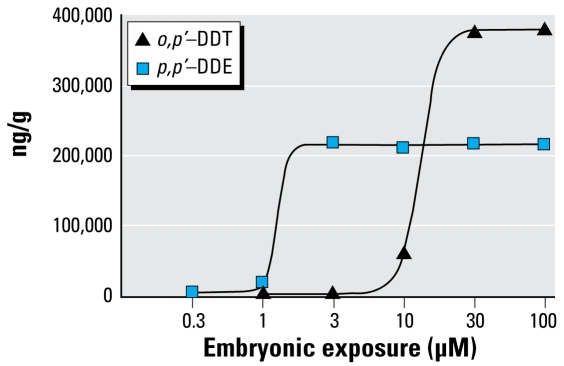
Amount (ng/wet weight) *o,p*′-DDT and *p,p*′-DDE detected in 7-dpf larvae as related to the initial bath embryonic exposure concentration.

Dose–response embryonic bath exposure to DDTs led to larval body burdens of *o,p*′-DDT and *p,p*′-DDE that reached maximal values at 375,000 and 215,000 ng/g tissue, respectively. (Figure 5). Both the 30 and 100 μM *o,p*′-DDT bath-exposed embryos reached this elevated concentration far above the other doses, contributing to the differences in formation and responses not seen in the other groups. The 10 μM *o,p*-′ DDT embryonic dose exhibited the most significant seizure response to PTZ and was selected as the dose for the domoic acid trials. This dose resulted in a larval body burden of 62,000 ng/g *o,p*′-DDT.

Based on the body burden values, the embryos seemed to uptake *p,p*′-DDE more readily then DDT. The 1-μM *p,p*′-DDE embryonic dose was chosen for domoic acid trials, as it exhibited a distribution of unique head-shake behaviors and defined seizure behaviors that could be quantified as increased mobility. This dose resulted in a body burden of 18,000 ng/g *p,p*′-DDE. The next-lower dose of 0.3 μM bath exposure, which led to a noticeable amount of PTZ-induced head-shake behavior, was carrying a body burden of 4,500 ng/g wet weight.

### Characterization of domoic acid seizure behavior in larval zebrafish

Untreated embryos were bath exposed to domoic acid concentrations as larvae (7 dpf) to catalog behavioral effects. Bath concentration of greater than 2 mM domoic acid resulted in mortality within an hour. Lower bath concentrations of 0.25–1.0 mM caused fish to express a brief hyperactive response prior to expression of convulsive behavior. The convulsions progressed, resulting in complete paralysis denoted by a lack of touch response and a reduction in heartbeats per minute. Classes were defined to categorize these specific behaviors based on perceived severity. Class 1 was a hyperactive response, charac terized by erratic swimming, operculum and jaw movements, and writhing or twitching motion. Class 2, convulsive behaviors, was dominated by body contractions, along with a circular swimming pattern created as the body experienced these motions. Class 3, paralysis, was noted when the fish stopped swimming and was oriented on its side or dorsal side down and confirmed with a lack of touch response. Closer examination at this stage showed the larvae had tremors isolated in the tail along with a labored heartbeat. The fish progressed through these behaviors faster as concentration increased. We chose a bath concentration of 0.36 mM for subsequent studies because it allowed the fish to progress through each seizure class rapidly without lethality.

### Effect of embryonic exposure to o,p′-DDT and p,p′-DDE on domoic acid–induced seizures in larval zebrafish

Because of the subtle behavioral characteristics exhibited by the domoic acid, the EthoVision software was unable to distinguish any differences either between different pretreatments or within the groups. The software scored many of the class 1 erratic behaviors the same as regular swimming, and other motions (tremors and smaller convulsions) were too small to be picked up by the TrackSys tower. Behavioral trials were subsequently observed under magnification and manually scored. Each incidence of behavior was recorded and grouped by class. Those fish exposed to organochlorines as embryos exhibited a distinct head-shake behavior when treated with domoic acid that was not observed in pretreatment controls. This behavior was in addition to class 2 seizure behavior, as it was frequently coupled with convulsive behaviors.

All embryos exhibited class 1 behaviors at some point during the trial with similar frequencies (Table 1). Embryonic organo-chlorine exposure resulted in faster progression to increased behavior severity. A majority of the class 1 behaviors were observed in the first 10 min after domoic acid exposure in the *o,p*′-DDT group, and from 10 to 20 min in controls. Both DDT and DDE exposure groups had all members reaching class 2 behaviors and a majority exhibiting class 3 symptoms (Table 1). Few controls reached these severe classes within 20 min but most had reached class 3 within 30 min, a time at which most organochlorine-exposed embryos were immobile. The severity of the class 2 behaviors was more intense in the organochlorine groups, as noted by the number of responses (Table 1). All DDT-exposed animals experienced a lack of touch response after 10 min of exposure (Table 1), further indicating the severity and progression of the behaviors.

## Discussion

### Design of zebrafish model relevant to wildlife exposure and disease

We investigated an embryonic basis to inducible seizure behavior in zebrafish under an experimental design to evaluate the potential for DDT enhancement of domoic acid toxicity. We found that body burdens of *p,p*′-DDE close to the levels found in fetal sea lions enhance seizure behavior to domoic acid. Previous studies have shown that immature mice on postnatal day 10 retain higher levels of DDT in brain after exposure and are about 100 times more sensitive to toxicity than adults (Eriksson 1984; Eriksson et al. 1990). The effect of DDT administered to these mice, just prior to completion of synaptogenesis, alters the development of muscarinic pathways in the cerebral cortex (Eriksson et al. 1992). Studies with additional environmental toxicants have established the general observation that a number of different agents disrupt neurodevelopment when administered during key milestones, which potentiates the reaction to toxicants later in life (Eriksson and Talts 2000). The study presented here not only provides similar findings using a zebrafish neurodevelopment model, but does so with an experimental design that sheds light on a wildlife population with well-defined dosage and temporal exposure to DDTs and domoic acid and also an emerging chronic juvenile neurologic disease (Goldstein et al. 2008; Goldstein et al., in press).

### Characterization of embryonic DDT exposure on PTZ-induced seizure behavior

We found that exposure of zebrafish embryos to subsymptomatic levels of the organochlorine pesticides *o,p*′-DDT and *p,p*′-DDE increases their sensitivity to the chemical convulsant PTZ during the larval period, a time when neurodevelopment is complete. Technical-grade DDT released into the environment is composed of approximately 80% *p,p*′ isomer and the remaining 20% *o,p*′ isomer. The active congener of DDT is metabo lized by reductive dehydrochlorination to the most stable and common form of DDE (Kitamura et al. 2002). DDTs are noted for their relatively high dose effects, causing a tremor syndrome in adult mice that includes hyperactivity, ataxia, and paralysis (Herr et al. 1985). This effect of DDT is due to a steric interaction with the inactivation of the voltage-gated sodium channel (Vijverberg et al. 1982), a molecular action found for both DDT isomers and *p,p*′-DDE (Rubin et al. 1993). Inhibition of channel inactivation causes a sodium ion leakage resulting in nerve hyperexcitibility and repetitive discharges. Although DDTs are also noted endocrine disruptors [DDT is a weak estrogenic agent (Bitman et al. 1968), and *p,p*′-DDE is a relatively potent antiandrogen (Kelce et al. 1995)], our findings of an increased seizure response to PTZ are consistent with a primary effect on voltage-gated sodium channels. Embryonic exposure to *o,p*′-DDT resulted in an increase in seizure severity to PTZ, as shown through increased distance traveled and increased time in a mobile state. Embryonic exposure to *p,p*′-DDE led to an increase mobility duration in response to PTZ with the appearance of previously unseen head-shaking behavior. Overall, these findings are consistent with the common finding that early-life exposure to toxicants enhances or alters responses to different toxicants later in life (Eriksson and Talts 2000).

### Evaluation of DTT body burden after embryonic exposure to DDTs

We next evaluated the body burden of these contaminants accumulated during embryogenesis in the larval zebrafish. Both *o,p*′-DDT and *p,p*′-DDE showed a curvilinear accumulation in the fish that reached a maximum at 375,000 and 215,000 ng/g tissue wet weight, respectively. The DDT whole-body burden leading to the strongest adverse effects for PTZ-induced seizures was 62,000 and 18,000 ng/g wet weight for *o,p*′-DDT and *p,p*′-DDE, respectively. Greater adverse effect levels for PTZ-induced seizures were still noted at lower DDT body burden of 6,000 and 4,500 ng/g wet weight for *o,p*′-DDT and *p,p*′-DDE, respectively. Given that *p,p*′-DDE is the predominant (91–99%) DDT congener in fetal sea lion blubber (Greig et al. 2007), the *p,p*′-DDE congener is most relevant to fetal sea lions to evaluate interaction with seizure-inducing agents.

**Figure 6:**
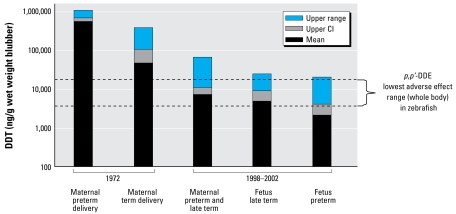
Concentration (ng/g wet weight) of total DDT found in maternal and fetal California sea lion blubber. Data from 1972 is modified from Gilmartin et al. (1976), and data from 1998–2002 is from Greig et al. (2007). The right side of the chart shows the relationship to the whole-body effect levels of *p,p*DDE (ng/g)-that enhance PTZ-induced seizures in zebrafish.

The adverse effect level we found for *p,p*′-DDE in the zebrafish is more than 3-fold less than the corresponding whole-body *p,p*′-DDE level (15,000 ng/g wet weight) of term fetuses of California sea lions modeled with 1991 data to consume (1,000 ng/g *p,p*′-DDE/wet weight) contaminated fish (Connolly and Glaser 2002). The *p,p*′-DDE body burden quantities that enhance seizure susceptibility in zebrafish are also useful in perspective to values in measured postpartum sea lions and their fetuses with consideration of the caveat that the sea lion values are blubber wet weight (Figure 6). The data show an order of magnitude reduction in DDT levels in postpartum sea lions over two decades. Fetal DDT levels in blubber were not reported in the earlier study; however, fetal DDT levels in 1998–2002 reached 20,000 ng/g wet weight in blubber. Factoring a low (5%) body fat at the time of birth, estimation of the whole-body *p,p*′-DDE of these fetal sea lions of 1998–2002 is about five times lower than the effect level we report in zebrafish. Although this upper estimate of *p,p*′-DDE in fetuses may correspond to a no-effect level to enhance PTZ toxicity, two additional factors (DDT levels several-fold higher in primiparous females and the potential for interaction with other accumulated organochlorines such as PCBs and polybrominated diphenyl ethers) further strengthen the case for interaction of fetal organochlorine burden on domoic acid toxicity.

### Characterization of embryonic DDT exposure on domoic acid–induced seizure behavior

The algal toxin domoic acid is a noted seizure-inducing agent in adult California sea lions (Gulland et al. 2002) and more recently has been associated with abortion and premature births of sea lions (Brodie et al. 2006; Goldstein et al., in press). Because sea lions are born with a mature nervous system, we tested the effect of domoic acid shortly after maturation of the nervous system of zebrafish at 7 dpf, a time used to characterize seizure response in zebrafish (Baraban et al. 2005). Domoic acid treatment of both *o,p*′-DDT and *p,p*′-DDE exposure groups had all members reaching class 2 seizure behaviors and a majority exhibiting class 3 seizure behaviors, whereas only a small percentage of zebrafish treated only with vehicle in embryogenesis showed these domoic acid–induced behaviors within the observation time. These results indicate that the early-life presence of DDTs, at body burdens relevant to those found in sea lions, shows increased susceptibility to domoic acid after completion of neurodevelopment.

### Relevancy of domoic acid exposure time for DDT-exposed embryos

The experimental design for this work used an embryonic zebrafish model to evaluate the potential effect of DDT body burden on *in utero* exposures to domoic acid of the California sea lion. Substantial investigation has focused on remarkably large transfers of maternal body burden of organochlorines by lactation in marine mammals (Addison and Brodie 1977, 1987). Yet, significant transfer also occurs during gestation, with DDTs accumulating in oocytes and continuing to transfer during embryogenesis and fetal growth. Research has shown that *p,p*′-DDE is one of the most common organochlorine contaminants found in human follicular fluid (Jarrell et al. 1993), and DDTs are carried in the plasma by lipoproteins (Mohammed et al. 1990) for transport to the follicle and entry into the oocyte via the oil droplet (Ungerer and Thomas 1996). Analysis of matched fetal and maternal blubber samples indicates substantial transplacental transfer of total DDT with ratios of 0.53 and 1.12 by wet weight and lipid weight, respectively (Greig et al. 2007). The majority of the DDT was present by midgestation, with mean wet weight ratios of 0.46 for preterm fetuses and 0.59 for term fetuses.

By immersing the zebrafish embryos from 6 to 30 hpf, the embryo absorbed the organo-chlorine during development between gastrulation and the scaffolding of the embryonic brain. Continued absorption of DDT into the embryo from that which accumulated in the yolk would be expected to continue through neurodevelopment. Exposure to seizure-inducing agents PTZ and domoic acid was performed at 7 dpf, when yolk absorption and neurodevelopment is largely complete. The 7-dpf zebrafish have been well characterized for seizure response to PTZ by behavioral, electrographic, and pharmacologic criteria and were determined to develop multiple phases of seizure activity mediated by glutamatergic:GABAergic neurotransmitter systems (Baraban et al. 2005). The PTZ and domoic acid symptomatology in 7-dpf zebrafish is comparable with symptoms observed in postnatal rodents exposed to these convulsive agents (Baraban et al. 2005). Allometric comparison of rodents to sea lions indicates that rat postnatal development corresponds to the final third of gestation of sea lion neurodevelopment, which in turn corresponds to the most common time for domoic acid poisoning of the California sea lion (Ramsdell and Zabka 2008). Hence, the exposure paradigm for this zebrafish model closely follows a likely exposure scenario for the California sea lion in which the organochlorine levels accumulate in the oocyte and continue through fetal life, and domoic acid exposure occurs later in fetal life when neuro-development is largely complete.

### Potential role of DTT and domoic acid in juvenile chronic disease in the California sea lion

Fetal toxicity of California sea lions has been observed as abortion and premature birthing and has been associated with several potential causative factors including organochlorine contamination, sea lion pathogens, and, more recently, domoic acid. This report has examined the interaction of two environmental toxicants, DDTs and domoic acid, given at exposure intervals during and after neurodevelopment, respectively, that are relevant to the California sea lion. Although it is possible that these agents may interact to cause abortion and premature birth, this work was directed instead to evaluate their role in the promulgation of adult disease. Recent observations have defined a chronic disease in juvenile California sea lions characterized by epilepsy and unusual behaviors (Goldstein et al. 2008). Given the increased sensitivity of the *in utero* period for domoic acid poisoning of the California sea lion and the delayed effects of *in utero* domoic poisoning in juvenile rodents (Dakshinamurti et al. 1993; Levin et al. 2005), this emerging chronic juvenile sea lion disease has been proposed to result from *in utero* toxicity to domoic acid (Ramsdell and Zabka 2008). The results presented here provide a basis for *p,p*′-DDE as an early developmental toxicant that enhances domoic acid poisoning of fetal California sea lions and the manifestation of chronic disease in juveniles.

## Figures and Tables

**Table 1:** Behavioral responses of 7-dpf zebrafish larvae to 0.36 mM domoic acid bath exposure grouped by classes of perceived increased severity. Class 1: hyperactivity; Class 2: convulsive behaviors; Class 3: no movement, on side, body tremors.

Domoic acid exposure		Class 1		Class 2		Class 3		
Embryonic treatment	Duration of exposure (min)	Individuals responding (%)	No. of responses	Individuals responding (%)	No. of responses	Individuals responding (%)	No. of responses	Absence of touch response (% of individuals)
Control/DMSO	0–5	62	15	8	1	8	4	0
	5–10	69	13	8	5	23	4	15
	10–15	85	24	15	3	8	1	15
	15–20	92	40	0	0	15	2	23
	0–20	100	92	23	9	38	11	23
*p,p*′-DDE (1 μM pretreated)	0–5	86	11	57	5	57	7	0
	5–10	43	18	43	11	43	10	14
	10–15	57	19	57	25	43	9	43
	15–20	86	26	100	20	71	17	86
	0–20	100	74	100	61	71	43	86
*o,p*′-DDT (10 μM pretreated)	0–5	100	59	100	47	86	26	43
	5–10	71	27	71	38	86	24	71
	10–15	14	2	43	19	71	18	100
	15–20	29	3	43	15	71	17	100
	0–20	100	91	100	119	100	85	100
